# Contribution of Piezo2 to endothelium-dependent pain

**DOI:** 10.1186/s12990-015-0068-4

**Published:** 2015-10-24

**Authors:** Luiz F. Ferrari, Oliver Bogen, Paul Green, Jon D. Levine

**Affiliations:** Division of Neuroscience, Department of Medicine, University of California, San Francisco, 521 Parnassus Avenue, San Francisco, CA 94143-0440 USA

**Keywords:** Piezo2, Stimulus-dependent hyperalgesia, Endothelin-1, Endothelial cell, Oxaliplatin

## Abstract

**Background:**

We evaluated the role of a mechanically-gated ion channel, Piezo2, in mechanical stimulation-induced enhancement of hyperalgesia produced by the pronociceptive vasoactive mediator endothelin-1, an innocuous mechanical stimulus-induced enhancement of hyperalgesia that is vascular endothelial cell dependent. We also evaluated its role in a preclinical model of a vascular endothelial cell dependent painful peripheral neuropathy.

**Results:**

The local administration of oligodeoxynucleotides antisense to Piezo2 mRNA, at the site of nociceptive testing in the rat’s hind paw, but not intrathecally at the central terminal of the nociceptor, prevented innocuous stimulus-induced enhancement of hyperalgesia produced by endothelin-1 (100 ng). The mechanical hyperalgesia induced by oxaliplatin (2 mg/kg. i.v.), which was inhibited by impairing endothelial cell function, was similarly attenuated by local injection of the Piezo2 antisense. Polymerase chain reaction analysis demonstrated for the first time the presence of Piezo2 mRNA in endothelial cells.

**Conclusions:**

These results support the hypothesis that Piezo2 is a mechano-transducer in the endothelial cell where it contributes to stimulus-dependent hyperalgesia, and a model of chemotherapy-induced painful peripheral neuropathy.

## Background

We have recently described a vascular endothelial cell-dependent, innocuous mechanical stimulus-induced enhancement of mechanical hyperalgesia that occurs following the administration of two potent vasoactive pronociceptive mediators, endothelin-1 and epinephrine [1, 2], but not other pronociceptive mediators (e.g., prostaglandin E_2_, nerve growth factor and tumor necrosis factor alpha) [1], at the site of nociceptive testing in the hind paw. In this innocuous stimulus-induced enhancement of mechanical hyperalgesia, after the local intradermal administration of endothelin-1 or epinephrine, repeated application of an innocuous mechanical stimulus produces a stepwise lowering of mechanical nociceptive threshold. Subsequent experiments demonstrated that endothelin-1 and epinephrine act at their cognate receptors on the endothelial cell and that following their administration at the site of nociceptive testing, innocuous mechanical stimuli induce the release of ATP from the endothelial cell, which in turn acts at P2X_2/3_, a ligand-gated ion channel receptor, on the peripheral terminal of the primary afferent nociceptor, to enhance mechanical hyperalgesia [3, 4].

A key feature of this stimulus-dependent hyperalgesia that remains to be established is the molecular basis of the transduction process for innocuous mechanical stimuli in the endothelial cell. Patapoutian and colleagues cloned a family of mechano-transducing ion channels, the Piezos that are gated by innocuous mechanical stimuli [5, 6]. More recently Patapoutian [7, 8], Gu [9] and colleagues, have demonstrated a key role of Piezo2 channels in the mechano-transduction process in Merkel cell-mediated touch. In the present study we provide evidence that Piezo2 contributes to endothelin-1-induced stimulus-dependent enhancement of mechanical hyperalgesia. In addition, since some neuropathic pain syndromes, such as that induced by oxaliplatin, affect vascular function [10–12], and vascular endothelium has a function in peripheral pain mechanisms [3, 4], we also evaluated the role of the endothelium and Piezo2 in a model of painful peripheral neuropathy produced by the cancer chemotherapy drug oxaliplatin.

## Results

### Contribution of peripheral Piezo2

**Fig. 1:**
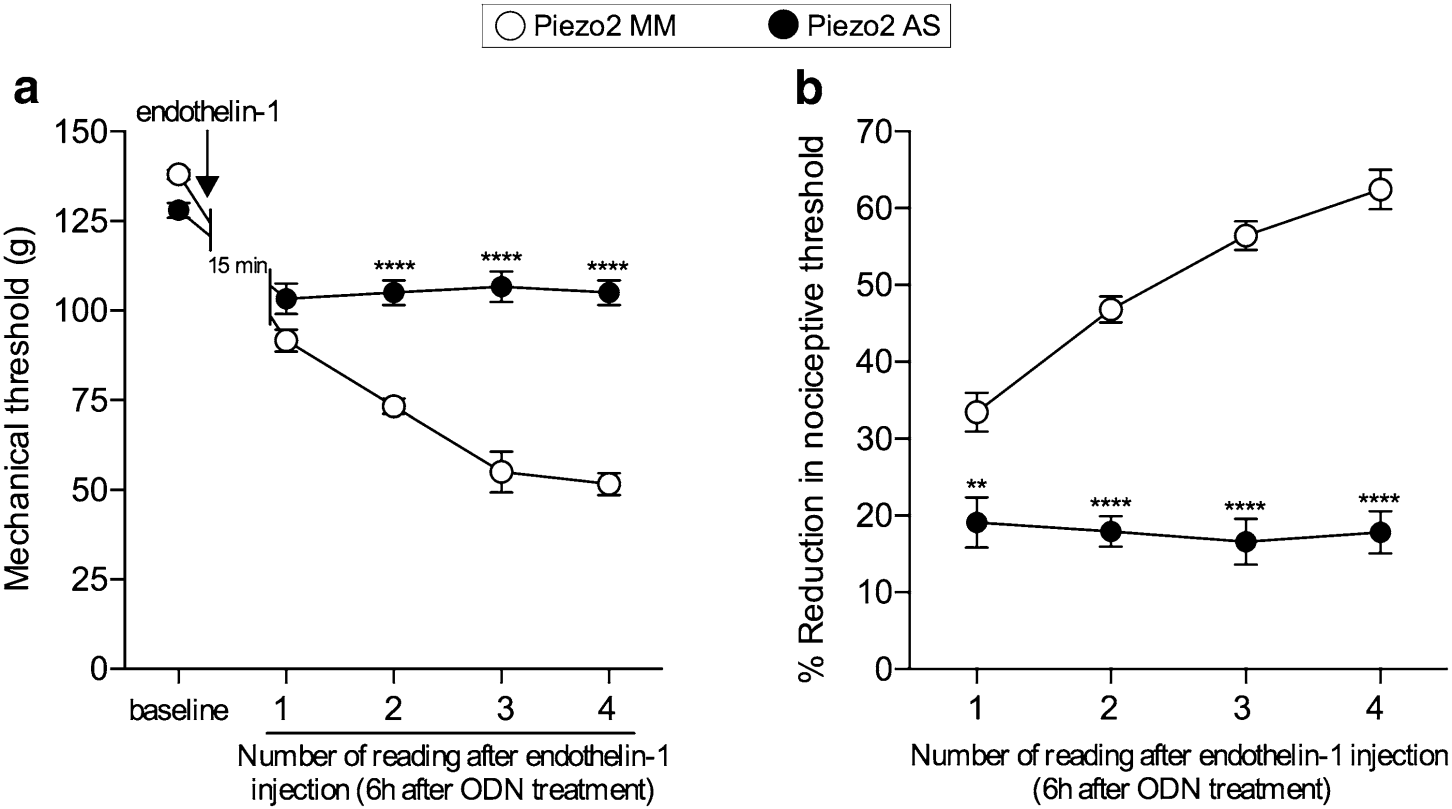
Role of Piezo2 in peripheral tissue in innocuous stimulus-induced enhancement of the mechanical hyperalgesia induced by endothelin-1. Rats received an intradermal injection of a combination of 3 sequences of ODN mismatch (MM, *open symbols*) or antisense (AS, *dark symbols*) against Piezo2 mRNA (20 μg of each sequence/μl, added to 2 μl of oligofectamine; total volume: 5 μl) on the dorsum of the hind paw. 6 h later, endothelin-1 (100 ng) was injected at the same site. At the same site, the paws then were submitted to 4 mechanical stimuli, 5 min apart from each other, starting 15 min after endothelin-1 injection. In the paws pretreated with local injection of ODN AS, compared to ODN MM, the decrease in the nociceptive threshold subsequent to the mechanical stimulations was significantly attenuated, indicating a role of peripheral Piezo2 channels in stimulus-dependent hyperalgesia. (F_1,10_ = 140.7; **p < 0.0061 and ****p < 0.0001, when the groups are compared at each reading, two-way repeated measures ANOVA followed by Bonferroni’s post hoc test; N = 6 paws per group). **a** Shows the mechanical nociceptive threshold, in grams, after each stimulation, starting 15 min post-endothelin-1 injection; in **b**, the reduction in the mechanical threshold after each stimulation, when compared to the baseline, is represented as percentage change

To evaluate the role of the Piezo2 channel in stimulus-dependent hyperalgesia, we injected a mixture of 3 oligodeoxynucleotide (ODN) sequences antisense against Piezo2 mRNA (see “Methods”), or of their mismatch ODNs, at the site of nociceptive testing on the dorsum of the rat’s hind paw (20 μg/1 μl for each sequence, added to 2 μl of oligofectamine, in a total volume of 5 μl, intradermally). Endothelin-1 (100 ng) was then injected at the same site, 6 h later. The mechanical nociceptive threshold was measured just prior to injection of endothelin-1 and then at 5-min intervals (X4), starting 15 min after the injection of endothelin-1. The administration of the Piezo2 antisense mixture, compared to mismatch, all but eliminated the sequential decrease in nociceptive threshold produced by each subsequent mechanical stimulus; that is, it eliminated stimulus-dependent hyperalgesia (Fig. 1). The residual hyperalgesia detected at the first test of nociceptive threshold is due to the action of endothelin-1 at the ET_A/B_ receptor(s) on the peripheral terminal of the nociceptor innervating the site of nociceptive testing [3]. These data support a role for Piezo2 as a mechano-transducer in endothelin-1-induced stimulus-dependent hyperalgesia.

### Non-neuronal location of peripheral Piezo2

**Fig. 2:**
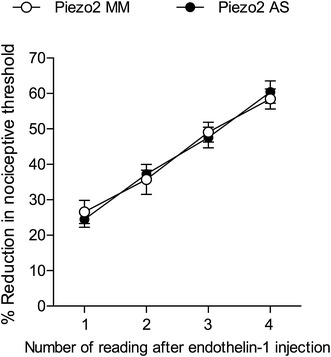
Piezo2 in sensory neurons innervating the skin do not play a role in endothelin-1-induced stimulus-dependent hyperalgesia. Rats were treated for 3 consecutive days with intrathecal injection of the combination of 3 sequences of ODN antisense (AS, *dark symbols*) or mismatch (MM, *open symbols*) against Piezo2 mRNA. On the 4th day, endothelin-1 (100 ng) was injected on the dorsum of the hind paw. Four mechanical stimuli, 5 min apart from each other, were performed, starting 15 min after endothelin-1 injection. No difference was observed between the groups in the decrease in the nociceptive threshold subsequent to the mechanical stimuli, indicating that Piezo2 channels in the DRG neurons are not involved in stimulus-dependent hyperalgesia induced by endothelin-1. (*p* = 0.9895, non-significant, when the groups are compared, two-way repeated measures ANOVA followed by Bonferroni’s post hoc test; N = 6 paws per group)

The Piezo2 channel has been demonstrated to be present in dorsal root ganglion (DRG) neurons [5]. Therefore, to rule out the possibility that the effect of intradermal Piezo2 ODN antisense was due to its uptake into the peripheral terminal of primary afferent nociceptors in the skin, followed by its transport to the cell body, and the subsequent down regulation of Piezo2 protein in the peripheral terminals of the primary afferents in the skin, we administered the same ODNs, antisense or mismatch, via the spinal intrathecal route of administration. However, unlike the intradermal administration of the Piezo2 ODN antisense, the intrathecal administration of the ODN antisense mixture against Piezo2 mRNA failed to attenuate stimulus-dependent hyperalgesia (Fig. 2). These findings support the suggestion that the ability of local peripheral administration of antisense to Piezo2 mRNA to attenuate stimulus-dependent hyperalgesia, is not due to its action on the primary afferent nociceptor, but rather by its action on Piezo2 containing cells in the skin.

### Role of Piezo2 in oxaliplatin-induced neuropathy

**Fig. 3:**
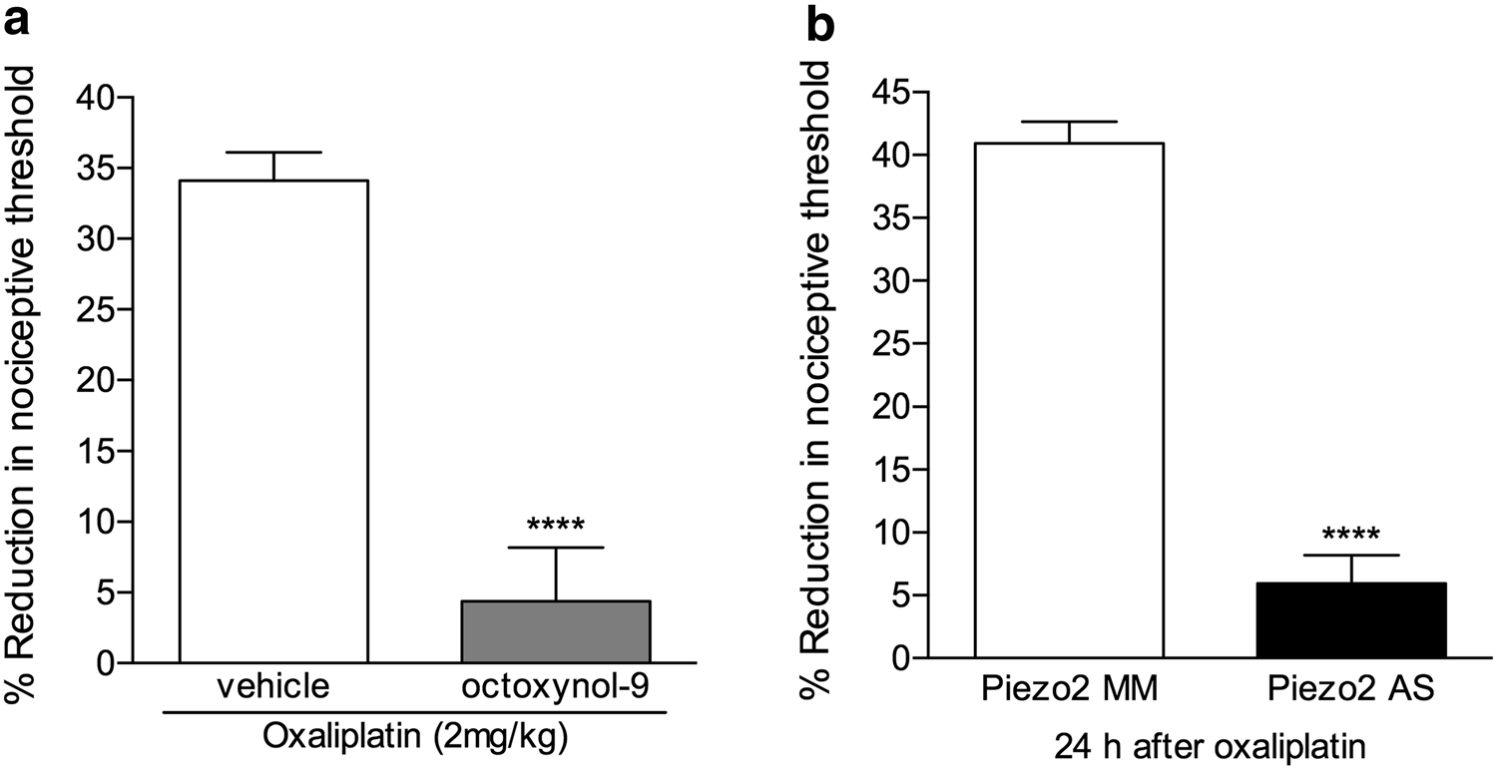
Role of the vascular endothelium and Piezo2 in peripheral tissue in oxaliplatin-induced neuropathic pain. **a** Rats that had been treated with a single intravenous injection of oxaliplatin (2 mg/kg) received, 48 h later, octoxynol-9 (0.5 % solution, injected intravenously; *gray bar*). The mechanical nociceptive thresholds were evaluated, by the Randall-Sellitto paw withdrawal test, 30 min after octoxynol-9 injection. We observed significant attenuation of the mechanical hyperalgesia induced by oxaliplatin in rats treated with octoxynol-9 when compared to a control group (*white bar*) (*t*_10_ = 6.923; ****p < 0.0001, when both groups are compared, Student’s *t* test), indicating a role of the endothelium in this model of chemotherapy-induced neuropathic pain (N = 6 paws per group); **b** rats received intravenous injection of oxaliplatin (2 mg/kg). 24 h later, the combination of 3 sequences of ODN mismatch (MM, *white bar*) or antisense (AS, *black bar*) against Piezo2 mRNA (20 μg of each sequence/μl, added to 2 μl of oligofectamine; total volume: 5 μl) was injected on the dorsum of the hind paw. 6 h later, the paws were submitted to 4 mechanical stimuli, 5 min apart from each other. We observed that in the paws treated with local injection of ODN AS, but not of ODN MM, the decrease in the nociceptive threshold subsequent to the mechanical stimuli was significantly attenuated, indicating a role of peripheral Piezo2 channels in oxaliplatin-induced hyperalgesia. (*t*_10_ = 12.38; ****p < 0.0001, when both groups are compared, Student’s *t* test; N = 6 paws per group)

In a preliminary experiment we observed that octoxynol-9, which attenuates endothelial cell function [13–15], attenuates mechanical hyperalgesia in oxaliplatin chemotherapy-induced painful peripheral neuropathy (unpublished data). To confirm a contribution of endothelial cells in oxaliplatin-induced painful peripheral neuropathy and evaluate for a role of Piezo2, we tested the effect of octoxynol-9 and Piezo2 antisense in our previously described model of oxaliplatin-induced hyperalgesia [16]. Intravenous injection of oxaliplatin (2 mg/kg) induced mechanical hyperalgesia that was significantly inhibited by intravenous injection of octoxynol-9 (Fig. 3a), which has been shown to impair endothelial function [13–15], implicating the endothelial cell in this model of cancer chemotherapy-induced painful peripheral neuropathy. It was not, however, possible to determine if there was a contribution of the mechanism underlying stimulus-dependent hyperalgesia or an alternative endothelial cell mechanism to the oxaliplatin-induced hyperalgesia, probably due to the degree of ongoing mechanical hyperalgesia. Hence, to evaluate if stimulus-dependent hyperalgesia contributes to the oxaliplatin-induced mechanical hyperalgesia, we attenuated its candidate mechano-transducer, Piezo2, using the local injection of the ODNs antisense to Piezo2 mRNA. We observed that the ODN antisense against Piezo2 mRNA injected at the site of nociceptive testing on the dorsum of the hind paw, strongly attenuated the mechanical hyperalgesia produced by oxaliplatin (Fig. 3b), compatible with the suggestion that the Piezo2 channel in endothelial cells plays a role in this form of neuropathic pain.

### Piezo2 mRNA in rat endothelial cells

**Fig. 4:**
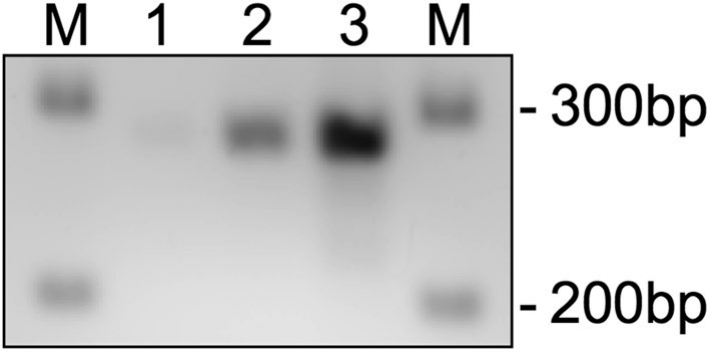
Expression of Piezo2 mRNA in endothelial cells. PCR analysis of Piezo2 mRNA was performed in extracts obtained from cultured rat aortic endothelial cells (RAOEC). cDNA from RAOEC was used to analyze whether endothelial cells express Piezo2. The size of the Piezo2 amplification product is 278 bp. *M* DNA—ladder; *1* 30 cycles, *2* 35 cycles; *3* 40 cycles

While the expression of Piezo2 in DRG neurons as well as in cells in the skin such as Merkel cells has been previously shown [7, 8, 17], its presence in the endothelial cell has not been studied. However, if Piezo2 plays a role in stimulus-dependent hyperalgesia, it must be expressed in the endothelial cell. Therefore, we determined if Piezo2 transcripts are present in rat endothelial cells, using PCR analysis on RNA extracts from rat aortic endothelial cells. As observed in Fig. 4, Piezo2 was detected in RNA extracts derived from cultured rat aortic endothelial cells.

## Discussion

While vascular pain syndromes are common [18–25] they are, unfortunately, often refractory to currently available analgesic therapy. This is due, at least in part, to a relative lack of understanding of the mechanisms of vascular pain, in comparison to pain arising from other structures. We recently described a novel, vascular endothelial cell-dependent mechanism for pain, a form of innocuous mechanical stimulation-induced release of pronociceptive mediators from endothelial cells that in turn sensitize nociceptors to intense mechanical stimuli, producing mechanical hyperalgesia. We have referred to this endothelial cell contribution to peripheral pain mechanisms as stimulus-dependent hyperalgesia [1, 4, 26]. In stimulus-dependent hyperalgesia, pronociceptive vasoactive substances such as endothelin-1 and epinephrine, acting at their cognate receptors on the endothelial cell, sensitize this cell to innocuous mechanical stimulus-induced release of a pronociceptive mediator, adenosine triphosphate (ATP) that in turn can act at P2X_2/3_ receptors to sensitize the peripheral terminal of the nociceptor, the mechanism underlying primary hyperalgesia [3, 4]. Importantly, with respect to the transduction properties of Piezo2 [7–9], innocuous stimuli are able to act at the endothelial cell to produce the ATP released in stimulus-dependent hyperalgesia [4].

We recently demonstrated a role of the vascular endothelial cell in preclinical models of persistent pain, including that induced by occupational exposure to intense low frequency high amplitude vibration and eccentric exercise [3] and in cancer chemotherapy-induced painful peripheral neuropathy produced by administration of oxaliplatin (unpublished data), a chemotherapy drug used to treat colorectal cancer [27–29]. The present study explored one of the key missing elements of the mechanism underlying stimulus-dependent hyperalgesia, the molecular basis of the mechanical transducer involved in the induction of the release of pronociceptive mediators from the endothelial cell by innocuous mechanical stimuli.

In the present experiments we tested the hypothesis that Piezo2, a mechanically-gated ion channel that is activated by relatively innocuous mechanical stimuli [8, 30, 31], could be the mechano-transducer in the endothelial cell that mediates the innocuous stimulus-induced enhancement of mechanical hyperalgesia induced by endothelin-1. In support of this hypothesis we found that ODNs antisense to Piezo2 mRNA, when injected intradermally at the site of nociceptive testing, but not intrathecally, at the site of the central terminal of the primary afferent nociceptor, which would be expected to attenuate expression of Piezo2 in the peripheral terminal of the nociceptor, eliminated stimulus-dependent hyperalgesia. These results support a role for Piezo2, in a cell in the skin, in stimulus-dependent hyperalgesia.

While there could be a cell in the skin, other than the endothelial cell, that contains Piezo2, which mediates stimulus-dependent hyperalgesia (e.g., Merkel cells [8]), the fact that stimulus-dependent hyperalgesia, which is eliminated by inhibition of vascular endothelial cell function [3, 26], is induced by two vasoactive pronociceptive mediators, endothelin-1 and epinephrine, whose receptors are present on endothelial cells, and is mediated by mechanical stimulus-induced release of pronociceptive mediators from the endothelial cell [4], supports the role of endothelial cell Piezo2 as the mechano-transducer responsible for stimulus-dependent hyperalgesia. However, since it has not previously been demonstrated that Piezo2 is present in endothelial cells, a necessary condition for endothelial cells to mediate innocuous stimulus-induced enhancement of endothelin-1 hyperalgesia, we examined for the presence of Piezo2 in endothelial cells. However, since endothelial cells constitute such a small percentage of cells in the skin, being a cellular monolayer lining the luminal surface of blood vessels, we addressed this question in cultured rat aortic endothelial cells. In this pure population of endothelial cells we were able to establish the presence of Piezo2 mRNA. To exclude a role of Piezo2 in the peripheral terminal of the nociceptor, we administered the Piezo2 antisense ODN intrathecally so that the only cell in the skin that could be affected by the antisense would be the peripheral terminal innervation of the sensory fibers the skin. This route of administration failed to attenuate stimulus-dependent hyperalgesia. While the pharmacology of Piezos in general, and Piezo2 in particular, are still relatively early in development, our data do support the suggestion that Piezo2 antagonists could be effective in the treatment of endothelial cell-dependent vascular pain syndromes. This is important since inhibiting ATP-dependent mechanisms, which mediate stimulus-dependent hyperalgesia [2–4], might have substantial adverse side effects.

Finally, to determine if Piezo2-dependent mechanisms in the skin contribute to an endothelial cell dependent preclinical pain syndrome, we evaluated the effect of the peripheral administration of ODNs antisense to Piezo2 mRNA in a preclinical model of a cancer chemotherapy-induced painful peripheral neuropathy, induced by oxaliplatin, which we found in preliminary experiments to be endothelial cell dependent. Attenuation of endothelial function, with octoxynol-9 or peripheral administration of ODN antisense to Piezo2 mRNA, both attenuated mechanical hyperalgesia in this model of chemotherapy-induced painful peripheral neuropathy, without affecting baseline paw withdrawal threshold in control rats. Thus, it would appear that stimulus-dependent sensitization can itself be so sensitized that it produces ongoing nociceptor sensitization in the absence of additional mechanical stimulation.

## Conclusions

**Fig. 5:**
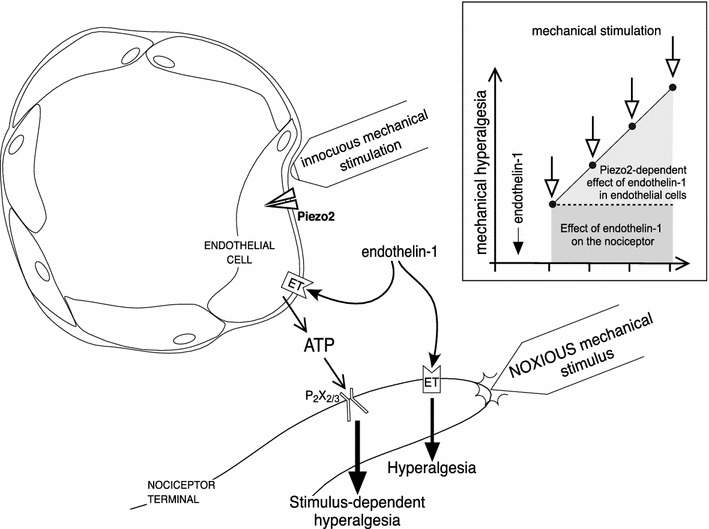
Schematic of vascular endothelial cell/Piezo2-dependent mechanism of innocuous stimulus induced enhancement of endothelin-1 hyperalgesia. Endothelin-1 activates ET receptors in the nociceptor terminal, sensitizing it to mechanical stimuli (hyperalgesia), detected as increased response to a noxious stimulus. The activation of ET receptors also sensitizes the endothelial cell to innocuous mechanical stimulation, inducing the release of ATP, which, in turn, act at P2X_2/3_ receptors on the nociceptor, producing enhancement of the endothelin-1-induced hyperalgesia (stimulus-dependent hyperalgesia). The insert represents the mechanical hyperalgesia induced by endothelin-1, acting on the nociceptor (*darker gray box*), and the increase in its magnitude after each stimulation (*open arrows*), due to a mechanism triggered by endothelin-1 at the endothelial cell (*lighter gray box*) involving Piezo2—which detects the innocuous mechanical stimulus—and the release of ATP

In summary, in this study we provide evidence that Piezo2 can function as a mechano-transducer in the endothelial cell, contributing to its role in vascular pain mechanisms, as described in the schematic in Fig. 5. However, which pain syndromes this mechanism contributes to remains to be established.

## Methods

### Animals

Experiments were performed on male Sprague–Dawley rats (200–250 g; Charles River). The experimental animals were housed three per cage, under a 12 h light/dark cycle, in a temperature- and humidity-controlled environment. Food and water were available to the rats ad libitum. All behavioral nociceptive testing was performed between 9:00 AM and 5:00 PM. Rats were acclimatized to the experimental environment and behavioral procedures before the actual experiments. To acclimate rats to the testing environment, they were brought to the room in which experiments were to be performed, in their home cages, where they were left for 15–30 min. After this they were placed in cylindrical transparent acrylic restrainers that have side vents that allow extension of the hind limbs from the restrainer for nociceptive testing. Rats were then left undisturbed in the restrainer for an additional 15–30 min before nociceptive testing was initiated. All experimental protocols were approved by the University of California, San Francisco, Committee on Animal Research, and conformed to National Institutes of Health *Guidelines for the Care and Use of Laboratory Animals*. Concerted effort was made to minimize the number of animals used and their suffering.

### Nociceptive testing

The nociceptive flexion reflex was quantified with an Ugo Basile Analgesymeter^®^ (Stoelting, Chicago, IL, USA), which uses a dome-shaped plinth to apply a linearly increasing mechanical force to the dorsum of the rat’s hind paw. Nociceptive threshold was defined as the force in grams at which the animal withdrew its paw. Using our previously established protocol for studying innocuous stimulus-induced enhancement of mechanical hyperalgesia [1, 26], single nociceptive threshold measurements were made prior to the intradermal injection of endothelin-1, and then 15, 20, 25, and 30 min after its administration. Hyperalgesia was defined as a decrease in mechanical nociceptive threshold, here presented as percentage decrease from baseline mechanical paw-withdrawal threshold. Each paw was treated as an independent measure; both paws of the same rat received the same treatment. Each experiment was performed on separate groups of rats.

### Drugs and reagents

The following drugs and reagents were used in this study: endothelin-1, oxaliplatin, and octoxynol-9, all obtained from Sigma-Aldrich (St. Louis, MO, USA). Endothelin-1 (100 ng) was dissolved in saline and administered intradermally on the dorsum of the hind paw, in a volume of 5 μl, using a 30-gauge hypodermic needle attached to a microsyringe (10 μl; Hamilton, Reno, NV, USA) by a short length of polyethylene tubing (PE-10). Oxaliplatin (dissolved in saline to a concentration of 2 mg/kg), used to induce painful peripheral neuropathy [16, 28, 32], and octoxynol-9 (0.5 % solution in saline, at a volume of 1 ml/kg), used to evaluate the contribution of the endothelium to nociceptive mechanisms [4, 13, 14], were administered intravenously via tail vein injection, followed by a bolus injection of an equal volume of saline before removal of the injection needle. Drug doses were selected based on the results of our previous studies [3, 4, 16, 33].

### ODN antisense sequences against Piezo2 mRNA

Because the endothelial cells that mediate stimulus-dependent hyperalgesia are a single cell layer thick lining in the dermal microvasculature, constituting an extremely small percentage of cutaneous tissue’s mass, we used an approach shown by Wood and colleagues to attenuate Piezo2 in more accessible cells, in the nervous system of the mouse [34]. In that study a mixture of 3 different ODN sequences all antisense to Piezo2 mRNA was used to attenuate the expression of Piezo2, demonstrating its functional role in sensory neurons [34]. Because of species differences between mouse and rat for the Piezo2 mRNA nucleotide sequence used by Wood and colleagues for their anti-Piezo2 antisense, we generated 3 nucleotide sequences designed based on the rat genome. The 3 sequences for the rat ODNs antisense to Piezo2 mRNA are: 5′-CCACCACATAAACACCTGC-3′, 5′-TTCCTCCTCTTCACTATCCG-3′ and 5′-CCTCAATGGTTTCCGTAGTTC-3′ (Invitrogen, Carlsbad, CA, USA), directed against three unique sequences of rat Piezo2 mRNA. The corresponding GenBank accession number and ODN position within the cDNA sequence are XM_255880.8 and 3740–3758, 5617–5636, 6238–6258, respectively. The ODN mismatch sequences, 5′-**A**CA**T**CACA**CG**AAC**T**CC**A**GC-3′, 5′-**G**TC**A**TC**G**TC**A**TCAC**AT**T**G**CG-3′ and 5′-**T**CTCA**G**TG**C**T**C**TCC**A**TAG**G**T**A**-3 correspond to the Piezo2 ODN antisense sequences with 6 or 7 bases mismatched (denoted by bold letters). To the mixture of the 3 antisense or mismatch mRNAs, 20 μg in a volume of 1 μl each, 2 μl of oligofectamine transfection reagent (Invitrogen) was added, completing a total volume of 5 μl, in order to facilitate the penetration of the ODNs into the endothelial cells. The ODNs were injected intradermally, on the dorsum of the rat’s hind paw. 6 h later endothelin-1 was administered at the same site. Of note, regarding the direct administration of the ODNs into the skin, the critical variable in this method is the half-life of Piezo2 in the endothelial cell, which is unknown. Among the different time points tested, 6 h was found to be long enough to observe a significant impact of the ODN antisense in our model. Therefore, we chose this time point to be used in the experiments shown in Figs. 1 and 3b. In separate groups of rats, the same doses (20 μg each) of the 3 ODNs, antisense or mismatch, were administered intrathecally. In this case, the ODNs were reconstituted in 0.9 % NaCl to a concentration of ~0.35 μg/μl, and administered daily for 3 consecutive days before the injection of endothelin-1 on the dorsum of the hind paw. During each injection, rats were anesthetized with 2.5 % isoflurane in 95 % O_2_. A 30-gauge hypodermic needle was then inserted, on the midline, into the subarachnoid space, between the L4 and L5 vertebrae. A total of 20 μl was slowly injected. The intrathecal site of injection was confirmed by a sudden tail flick, a reflex that is evoked by subarachnoid space access and bolus injection [35]. Animals regained consciousness approximately 1 min after the injection. The use of antisense to attenuate the expression of proteins in nociceptors, important for their role in nociceptor sensitization, is well supported by previous studies by others [36–39], as well as our group [40–43].

### Polymerase chain reaction (PCR)

Total RNA from cultured rat aortic endothelial cells (RAOEC, Cell Applications, San Diego, CA, USA) was extracted using Trizol reagent (Invitrogen/Thermo Fisher Scientific, Waltham, MA, USA) with the PureLink™ RNA mini kit (Ambion/Thermo Fisher Scientific, Waltham, MA, USA) according to the manufacturer’s instruction. The amount of RNA was determined with an UV-spectrophotometer and 1 µg of RNA was used for cDNA synthesis using the SuperScript III Platinum One-Step quantitative RT-PCR System (Invitrogen/Thermo Fisher Scientific, Waltham, MA, USA). 10 µl of the cDNA were used to analyze for Piezo2 expression. The PCR-primers used for the amplification of rat Piezo2 were: F1: 5′-ACACCATGCTGGTGCTCATC-3′ and B1: 5′-AGGGTGGGCTAACCTGTAGA-3′ and matched against NCBI database-entry XM_225880. The Piezo2 PCR products were electrophoretically separated on a 2 % agarose gel and visualized by ethidium bromide intercalation.

### Statistical analyses

The dependent variable in these experiments was change in paw withdrawal threshold, expressed as percentage, or in Fig. 1a also absolute change in grams, from the pretreatment baseline threshold. Group data are represented as mean ± SEM. Statistical significance was determined by two-way repeated-measures ANOVA, followed by Bonferroni’s post hoc test, or by Student’s *t* test (as noted in the Figure Legends). *p* values <0.05 were considered statistically significant.

## Abbreviations

AS: antisense
DRG: dorsal root ganglion
MM: mismatch
ODN: oligodeoxynucleotide
SEM: standard error of the mean
